# Identification of the EBF1/ETS2/KLF2-miR-126-Gene Feed-Forward Loop in Breast Carcinogenesis and Stemness

**DOI:** 10.3390/ijms26010328

**Published:** 2025-01-02

**Authors:** Alessandra Gambacurta, Valentina Tullio, Isabella Savini, Alessandro Mauriello, Maria Valeria Catani, Valeria Gasperi

**Affiliations:** 1 Department of Experimental Medicine, Tor Vergata University of Rome, 00133 Rome, Italy; gambacur@uniroma2.it (A.G.); valentinatullio.nu@gmail.com (V.T.); savini@uniroma2.it (I.S.); alessandro.mauriello@uniroma2.it (A.M.); 2 NAST Centre (Nanoscience & Nanotechnology & Innovative Instrumentation), 00133 Rome, Italy

**Keywords:** bioinformatics, breast cancer, epigenetics, microRNAs, neural stemness, transcription factors

## Abstract

MicroRNA (miR)-126 is frequently downregulated in malignancies, including breast cancer (BC). Despite its tumor-suppressive role, the mechanisms underlying miR-126 deregulation in BC remain elusive. Through silencing experiments, we identified Early B Cell Factor 1 (EBF1), ETS Proto-Oncogene 2 (ETS2), and Krüppel-Like Factor 2 (KLF2) as pivotal regulators of miR-126 expression. These transcription factors were found to be downregulated in BC due to epigenetic silencing or a “poised but not transcribed” promoter state, impairing miR-126 expression. Gene Ontology analysis of differentially expressed miR-126 target genes in the Cancer Genome Atlas: Breast Invasive Carcinoma (TCGA-BRCA) cohort revealed their involvement in cancer-related pathways, primarily signal transduction, chromatin remodeling/transcription, and differentiation/development. Furthermore, we defined interconnections among transcription factors, miR-126, and target genes, identifying a potential feed-forward loop (FFL) crucial in maintaining cellular identity and preventing the acquisition of stemness properties associated with cancer progression. Our findings propose that the dysregulation of the EBF1/ETS2/KLF2/miR-126 axis disrupts this FFL, promoting oncogenic transformation and progression in BC. This study provides new insights into the molecular mechanisms of miR-126 downregulation in BC and highlights potential targets for therapeutic intervention. Further research is warranted to clarify the role of this FFL in BC, and to identify novel therapeutic strategies aimed at modulating this network as a whole, rather than targeting individual signals, for cancer management.

## 1. Introduction

MicroRNA (miR)-126, a member of the small noncoding RNA family, holds a pivotal role in governing gene expression [1]. Localized within intron 9 of the *epidermal growth factor-like-domain 7* (*EGFL7*) gene, miR-126 is intertwined with the regulation of endothelial cell function, specifically in orchestrating angiogenesis [1]. As master regulator of physiological angiogenesis and vascular integrity, miR-126 is usually deregulated in angiogenesis-related disorders, thus compromising the vascular integrity and functional properties of endothelial progenitor cells [2,3].

Moreover, over the past decade, aberrant miR-126 expression has surfaced in diverse pathologies, encompassing Alzheimer’s disease, autoimmune and liver disorders [4,5,6], and cancer. In particular, miR-126 is usually downregulated in tumors [7,8,9,10,11,12,13] and, by regulating specific gene networks, it mainly acts as a tumor suppressor, as documented in glioma, non-small cell lung, pancreas, colon, and breast cancers [11,12,14,15,16,17,18,19,20,21,22].

Despite extensive research, mechanisms driving miR-126 downregulation in cancer remain enigmatic. Recent focus has turned to DNA methylation alterations underlying the downregulation of miR-126 expression. For instance, silencing of the *EGFL7/miR-126* gene, via promoter hypermethylation, has been described for lung, glioma, mesothelioma, colorectal cancers, and esophageal squamous cell carcinoma [23,24,25,26,27,28,29,30]. Nonetheless, low DNA methylation levels observed in other cancer types, including bladder and prostate tumors [10], suggest alternative regulatory mechanisms beyond DNA methylation. Notably, despite miR-126 involvement in breast cancer (BC) [11,15,31], to date, the molecular mechanisms underlying miR-126 expression in BC remain unexplored.

Based on this background, by using a multi-faceted approach encompassing bioinformatics, and in vitro and ex vivo studies, we investigated the mechanisms responsible for miR-126 expression, shedding light on alternative upstream event(s) driving its downregulation in BC.

## 2. Results

### 2.1. EGFL7/miR-126 Downregulation in BC

**Figure 1:**
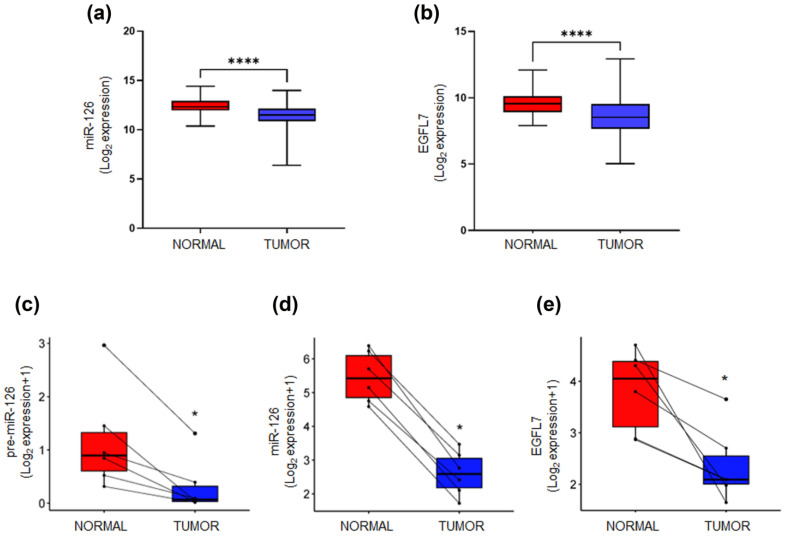
MiR-126 and EGFL7 expression in breast cancer. Expression of (**a**) miR-126 and (**b**) EGFL7 in normal and breast tumor samples from TCGA-BRCA RNA-seq and miRNA-seq data. For miR-a126, normal = 104 and tumor = 1078. For EGFL7, normal = 112 and tumor = 1093. (**c**) pre-miR-126, (**d**) miR-126, and (**e**) EGFL7 expression analysis, by qRT-PCR, in luminal breast tumor and matched-normal biopsies (n = 6). Solid black lines illustrate expression of pre-miR-126, miR-126 and EGFL7 for each patient. Each box plot with the whiskers indicates the median, maximum, and minimum expression value, and data are reported either as Log_2_ expression (for TCGA-BRCA) or Log_2_ expression + 1 (for human biopsies). **** *p* < 0.0001 versus normal samples, calculated by Mann–Whitney test. * *p* = 0.0313 versus matched-normal tissues, calculated by Wilcoxon matched-pairs signed rank test.

We first assessed expression levels of miR-126 and its host gene EGFL7, in human BC tissues using data from The Cancer Genome Atlas: Breast Invasive Carcinoma (TCGA-BRCA) database. Our bioinformatic analysis documented a significant downregulation of both miR-126 (Figure 1a) and EGFL7 (Figure 1b) in BC samples compared to non-neoplastic tissues (*p* < 0.0001 for both markers). However, this did not clarify whether this dysregulation was due to altered biogenesis and/or post-transcriptional events.

We then conducted a case–control study to analyze the expression profiles of pre-miR-126, miR-126, and EGFL7 in BC and matched adjacent tissues, from six female patients diagnosed with luminal BC at Policlinico Tor Vergata of Rome. All tumor biopsies showed significantly lower expression of pre-miR-126 compared to adjacent normal counterparts (*p* < 0.005) (Figure 1c). Similarly, miR-126 and EGFL-7 levels were also lower in cancer tissues compared to paired normal tissues (Figure 1d,e).

### 2.2. Epigenetic Profiling of EGFL7/miR-126 Gene

To explore the epigenetic mechanisms behind EGFL7/miR-126 downregulation, we examined DNA methylation and histone modifications associated with active (H3K4me3, H3K27ac, and H3K36me3) or repressed (H3K27me3) gene transcription [32,33,34], as well as RNA polymerase II (RNA Pol II) occupancy and associated RNA-seq. Data for normal and tumor breast samples were visualized by the Integrative Genomics Viewer (IGV) browser [35]. We analyzed the three experimentally verified *EGFL7* promoters and a putative promoter region suggested to control miR-126 expression in an EGFL7-independent manner (promoters I–IV) (Supplementary Figure S1) [2,36].

Unlike the literature data reporting DNA hypermethylation as responsible for EGFL7/miR-126 downregulation in various tumors [23,24,25,26,27,28,29], we found the chromosomal region spanning the *EGFL7/miR-126* gene to be hypomethylated in BC (Supplementary Figure S1a). In normal tissue, histone marks indicated an open state of the *EGFL7/miR-126* gene, with high levels of H3K4me3, H3K36me3, and H3K27ac associated with low levels of H3K27me3 (Supplementary Figure S1b). RNA-seq confirmed mRNA transcription starting from promoter II (Supplementary Figure S1b). Tumor cells displayed a similar open state (Supplementary Figure S1b), but RNA Pol II occupancy and RNA-seq showed transcription starting from promoter III (Supplementary Figure S1b). This result, confirmed by ATAC-seq and GRO-seq analysis, denoted the expression of a shorter *EGFL7/miR-126* transcript (Supplementary Figure S1c). Overall, there were no significant differences in the epigenetic profile between tumor and normal breast.

### 2.3. Regulation of EGFL7/miR-126 Transcription by EBF1, ETS2, and KLF2

**Figure 2:**
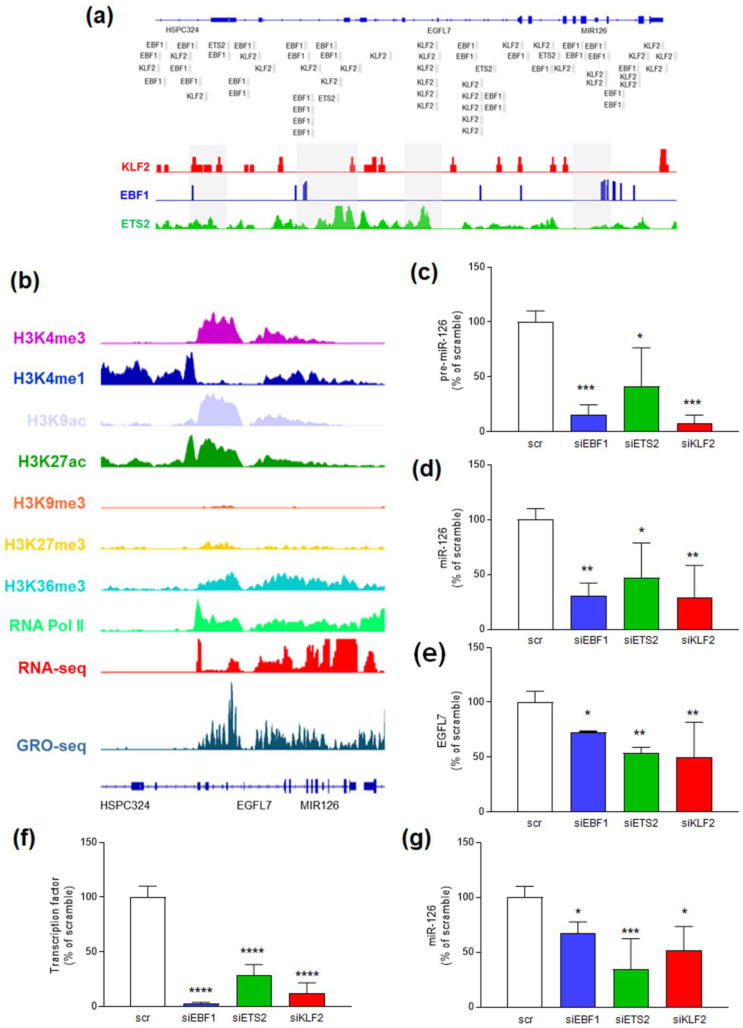
Involvement of KLF2, EBF1, and ETS2 TFs in *EGFL7/miR-126* transcription. (**a**) Genome binding/occupancy profiling of KLF2, EBF1, and ETS2 on the *EGFL7/miR-126* gene. Upper panel: Jaspar Core 2024 tracks showing predicted binding sites for the three TFs along the *EGFL7/miR-126* gene, visualized by UCSC Genome browser. Lower panel: IGV visualization of ChIP-seq datasets relative to KLF2 (red), EBF1 (blue), and ETS2 (green) binding sites in the promoter regions (highlighted in grey) of the *EGFL7/miR-126* gene. (**b**) IGV visualization of active (H3K4me1, H3K4me3, H3K9ac, H3K27ac, and H3K36me3) and repressive (H3K27me3 and H3K9me3) histone modifications, RNA Pol II occupancy, RNA-seq and GRO-seq of the *EGFL7/miR-126* gene, in HUVEC cells. (**c**) pre-miR-126, (**d**) miR-126, and (**e**) EGFL7 expression, in HUVECs, analyzed by qRT-PCR, after 48 h transient transfection with either KLF2 (red) or EBF1 (blue) or ETS2 (green) siRNAs. (**f**) EBF1, ETS2, KLF2 and (**g**) miR-126 expression in HMECs, analyzed by qRT-PCR, after 48 h transient transfection with either EBF1 (blue) or ETS2 (green) or KLF2 (red) siRNAs. Values are reported as percentage of scramble-transfected cells (scr), arbitrarily set to 100%. Data are shown as mean ± S.D. of three independent experiments, each performed in triplicate. * *p* < 0.05, ** *p* < 0.01, *** *p* < 0.005, and **** *p* < 0.001 versus scramble, calculated by Dunnett’s multiple comparisons test.

We next investigated whether specific transcription factors (TFs) were involved in EGFL7/miR-126 downregulation. We focused on KLF2 (Krüppel-Like Factor 2), ETS2 (ETS Proto-Oncogene 2), and EBF1 (Early B Cell Factor 1), which are usually deregulated in several cancers, including BC [37,38,39,40,41,42], and have several putative binding sites on the *EGFL7* gene (Figure 2a) [36,43]. ChIP-seq analysis showed KLF2 binding to promoters I and II, EBF1 to promoters I, II, and IV, and ETS2 to all promoter regions (Figure 2a).

The miR-126 specificity towards endothelial cells, where it acts as a master regulator of angiogenesis and vascular integrity [44,45], prompted us to perform silencing experiments of the three TFs in Human Umbilical Vein Endothelial Cells (HUVECs). These cells, indeed, express high levels of miR-126 and EGFL7 [44], as confirmed by the open chromatin state of the *EGFL7/miR-126* gene and by RNA-seq and GRO-seq data (Figure 2b). Silencing experiments using siRNAs specific for each TF (Supplementary Figure S2) showed that knockdown of these TFs significantly reduced pre-miR-126 expression, with KLF2 and EBF1 siRNAs being the most effective (*p* < 0.005 compared to scramble transfected cells) (Figure 2c). A similar trend was observed for miR-126 expression (Figure 2d), and all siRNAs reduced EGFL7 mRNA levels by about 40% (Figure 2e).

To understand if these findings could be translated in BC, we carried out the same experiments on primary Human Mammary Epithelial Cells (HMECs) (Figure 2f,g). Like in HUVEC cells, EBF1, ETS2, and KLF2 silencing led to significant reduction in miR-126 expression, with KLF2 and ETS2 siRNAs being the most effective (Figure 2f,g). Collectively, our data confirmed that EBF1, ETS2, and KLF2 are all involved in *EGFL7/miR-126* transcription.

### 2.4. Downregulation of EBF1, ETS2, and KLF2 in BC

We then analyzed EBF1, ETS2, and KLF2 expression in BC using the TCGA-BRCA dataset. Among 1205 RNA-seq samples, we selected 112 matched-paired luminal BC samples and further restricted to 73 BC individuals with both mRNA-seq and miRNA-seq data. EBF1, KLF2, and ETS2, along with EGFL7 and miR-126, were significantly downregulated in BC specimens compared to healthy tissues (Supplementary Figure S3a). Accordingly, in our tumor specimens, EBF1 and ETS2 were significantly lower than their matched normal counterparts, while KLF2 showed no significant differences (Supplementary Figure S3b,c). Nevertheless, consistency between our qRT-PCR results and TCGA-BRCA data (Supplementary Figure S3d) indicated that our patient cohort was representative, despite the low sample size.

Pearson correlation analysis of TCGA-BRCA data showed miR-126 expression positively correlated with EBF1 (r = 0.5194, *p* < 0.0001) and KLF2 (r = 0.3117, *p* = 0.0073), but not with ETS2; KLF2 also strongly positively correlated with both EBF1 (r = 0.6822, *p* < 0.0001) and ETS2 (r = 0.5362, *p* < 0.0001) (Supplementary Figure S4a–f). A principal component analysis (PCA) biplot revealed that tumor patients had lower expression of the five genes, distinguishing them from healthy individuals (Supplementary Figure S4g); therefore, discrimination between healthy and tumor samples appeared to depend on overall gene expression pattern rather than one individual gene (Supplementary Figure S4g).

**Figure 3:**
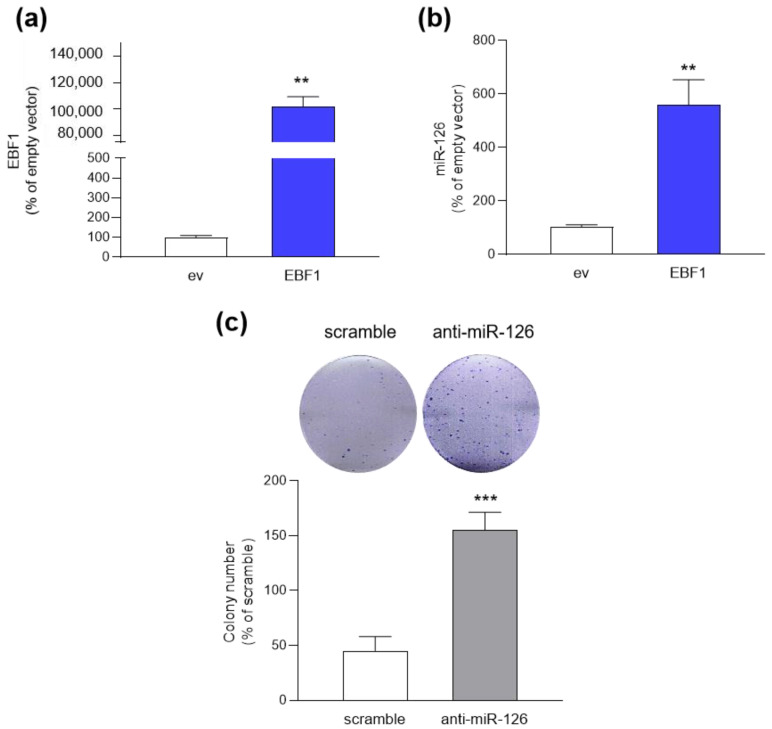
Modulation of miR-126 levels in luminal BC and healthy mammary epithelial cells. (**a**) EBF1 expression in MCF-7 cells, analyzed by qRT-PCR, after 48 h transient transfection with either pCMV6-AC empty vector (ev) or pCMV6-AC-EBF1 (EBF1). (**b**) miR-126 expression in EBF1-overexpressing MCF-7 cells. Cells were treated as in (**a**) and analyzed by qRT-PCR. (**c**) CFU assay performed with HMECs transiently transfected with either scramble oligo (scramble), or miR-126-inhibitor (anti-miR-126) for 48 h. Photographs refer to the wells of a 6-well plate and are representative of three independent experiments. Histograms show colony number reported as percentage of scramble, arbitrarily set to 100% (absolute colony number = 45.17 ± 12.84). Data are shown as mean ± SD. ** *p* = 0.0079 and *** *p* = 0.0022 versus relative control [ev in (**a**,**b**); scramble-transfected cells in (**c**)], calculated by Mann–Whitney test.

The correlation between TFs and miR-126 in BC was experimentally confirmed. In the luminal MCF-7 BC cell line, EBF1 overexpression (Figure 3a) resulted in a six-fold increase in miR-126 expression (Figure 3b). Conversely, in HMEC cells, miR-126 inhibition, by transfection with anti-miR-126 oligo, considerably enhanced colony-forming ability by three-fold (Figure 3c). These observations confirmed the regulatory role of EBF1 in controlling miR-126 levels within the context of BC and that miR-126 plays a role in suppressing cell proliferation and potentially tumorigenesis in normal breast epithelial tissue.

### 2.5. Epigenetic Profiling of EBF1, ETS2, and KLF2

To understand the regulatory mechanisms behind the downregulation of EBF1, ETS2, and KLF2 in BC, we investigated their epigenetic landscape.

**Figure 4:**
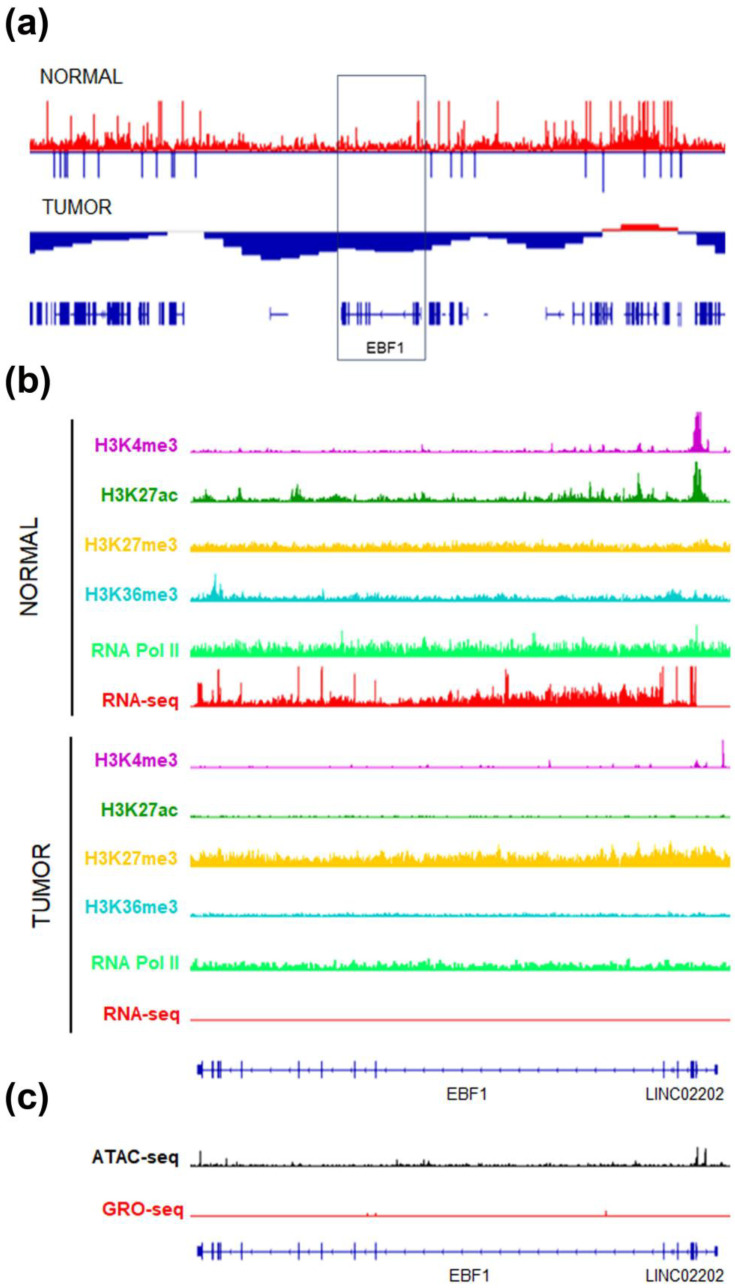
*EBF1* gene epigenetic signatures in normal and tumor breast samples. (**a**) DNA methylation (blue) and DNAase hypersensitive sites (red) in the genome region containing the *EBF1* gene (box), in normal and tumor breast samples. (**b**) IGV visualization of histone modifications, RNA Pol II and RNA seq data of the *EBF1* gene in normal and tumor luminal breast samples. (**c**) IGV tracks showing ATAC-seq and GRO-seq in tumor luminal breast samples.

In normal luminal epithelium, the *EBF1* gene exists in an open chromatin state, characterized by DNA hypomethylation and active histone marks such as H3K4me3, H3K27ac, and H3K36me3 (Figure 4a,b). This epigenetic configuration supports active transcription, which was confirmed by RNA Pol II occupancy and RNA-seq data, showing a “not paused and transcribed” pattern for EBF1 (Figure 4b). Conversely, breast tumor displayed an overall repressed state of the *EBF1* gene, as indicated by DNA hypermethylation (Figure 4a), and repressive H3K27me3 modification (Figure 4b). The lack of transcriptional activity in tumors was further validated by the absence of RNA Pol II binding and EBF1 transcripts in RNA-seq and GRO-seq analyses (Figure 4b,c).

**Figure 5:**
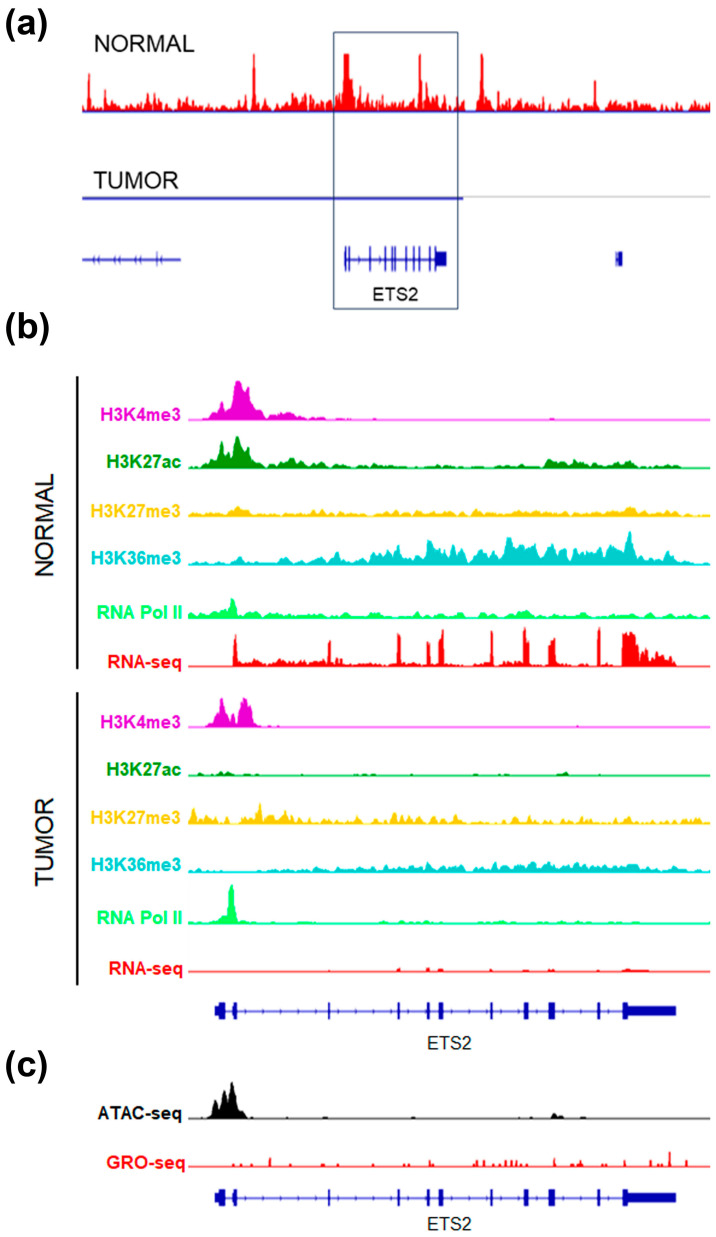
*ETS2* gene epigenetic signatures in normal and tumor breast samples. (**a**) DNA methylation (blue) and DNAase hypersensitive sites (red) in the genome region containing the *ETS2* gene (black box), in normal and tumor breast samples. (**b**) IGV visualization of histone modifications, RNA Pol II and RNA seq data of the *ETS2* gene in normal and tumor luminal breast samples. (**c**) IGV tracks showing ATAC-seq and GRO-seq in tumor luminal breast samples.

Similarly, the *ETS2* gene in normal breast epithelium displayed an open chromatin state, marked by DNA hypomethylation and active histone modifications. Transcriptional activity was confirmed by RNA Pol II binding and RNA-seq data (Figure 5a,b). However, in breast tumor cells, despite the promoter being accessible as indicated by H3K4me3 marks, ATAC-seq, and RNA Pol II occupancy, *ETS2* transcription was paused. This was evidenced by the almost complete absence of ETS2 transcripts in RNA-seq and GRO-seq analyses (Figure 5b,c).

**Figure 6:**
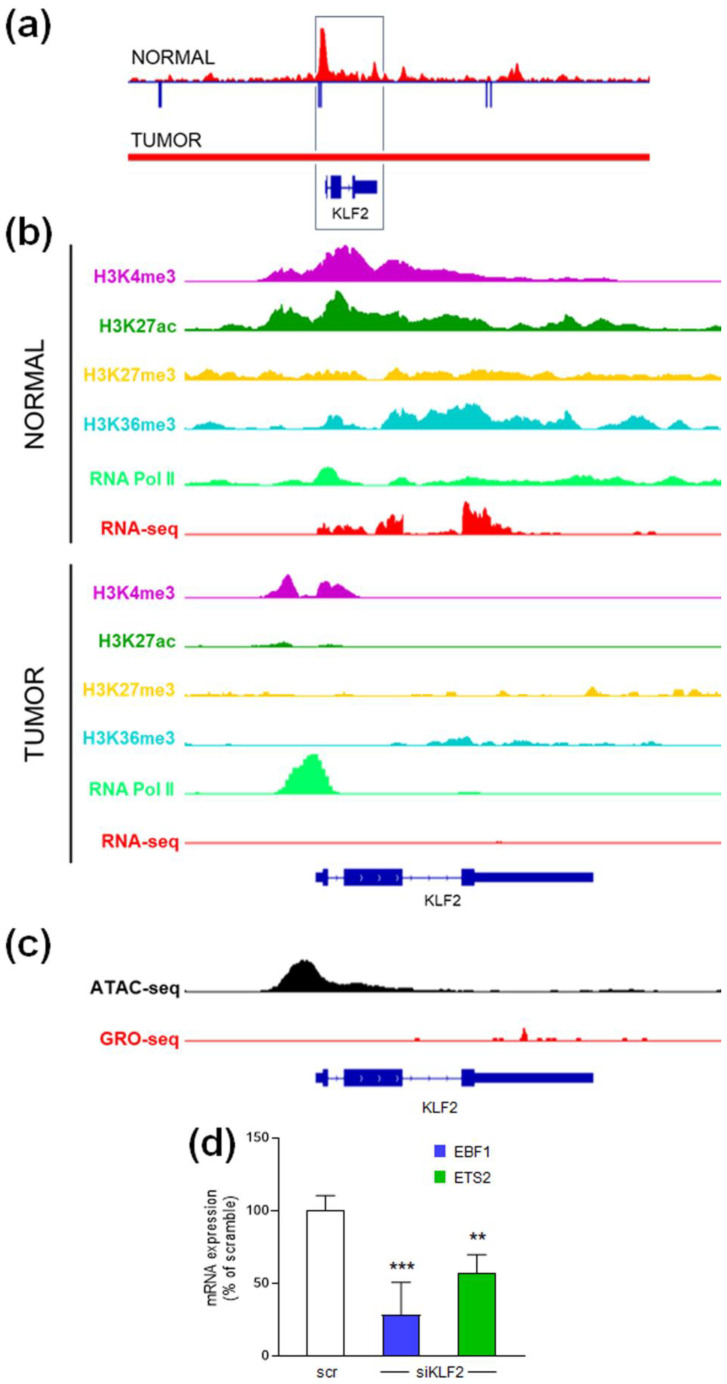
*KLF2* gene epigenetic signatures and KLF2-dependent regulation of EBF1 and ETS2 expression. (**a**) DNA methylation (blue) and DNAase hypersensitive sites (red) in the genome region containing the *KLF2* gene (black box), in normal and tumor breast samples. (**b**) IGV visualization of histone modifications, RNA Pol II and RNA seq data of the *KLF2* gene in normal and tumor luminal breast samples. (**c**) IGV tracks showing ATAC-seq and GRO-seq in tumor luminal breast samples. (**d**) EBF1 and ETS2 expression, analyzed by qRT-PCR, in HUVEC cells, after 48 h transient transfection with KLF2 siRNA. Values are reported as percentage of scramble-transfected cells (scr), arbitrarily set to 100%. Data are shown as mean ± S.D. of three independent experiments, each performed in triplicate. ** *p* < 0.01 and *** *p* < 0.001 versus scramble, calculated by Dunnett’s multiple comparisons test.

The *KLF2* gene also exhibited an open chromatin state in both normal and tumor tissues, as indicated by DNA hypomethylation and active histone marks (Figure 6a,b). In normal cells, *KLF2* transcription was active, shown by RNA Pol II binding and RNA-seq data. In tumor cells, although the promoter remained accessible and RNA Pol II was present, the transcription was paused, as demonstrated by the lack of KLF2 transcripts in RNA-seq and GRO-seq analyses (Figure 6b,c).

The apparent discrepancy between the epigenetic profile of KLF2 and the results from silencing experiments prompted us to explore the interrelationship between KLF2 and the other two TFs. We discovered that EBF1 and ETS2 genes contain predicted binding sites for KLF2Functional validation through KLF2 knockdown experiments in HUVECs showed a significant reduction in the expression of EBF1 (71%) and ETS2 (43%) (Figure 6d). This suggests that KLF2 plays a regulatory role in miR-126 expression not only directly but also indirectly by modulating *EBF1* and ETS2 transcription.

### 2.6. Identification of a TF-miR-126-Gene Feed-Forward Loop

**Figure 7:**
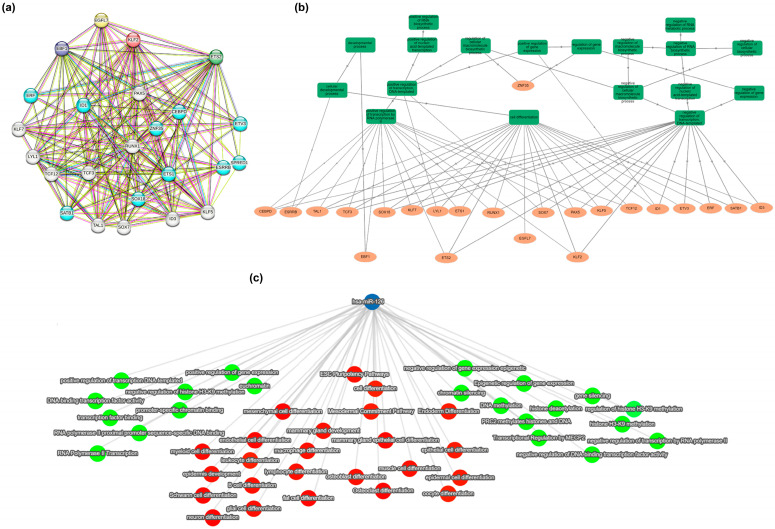
Transcription factor/miR-126 networks. (**a**) STRING analysis of EBF1/ETS2/KLF2/EGFL7 interacting networks. Colored nodes: query proteins and first shell of interactors; white nodes: second shell of interactors. Light blue line: database evidence; purple line: experimental evidence; green line: neighborhood evidence; blue line: co-occurrence evidence; red line: gene fusion evidence; yellow line: text mining evidence; black line: co-expression evidence; violet line: protein homology. (**b**) Hierarchical view of biological processes (BPs) related to genes shown in (**a**), by GOnet (*p* value threshold < 0.00 × 10^0^). Squares: categories. Circles: genes. (**c**) Selected BP interaction network of miR-126 by miRTargetLink 2.0. Blue circle: miR-126; red circles: BP connected to cell differentiation; green circles: BP connected to either negative or positive regulation of gene expression.

To delve deeper into the potential regulatory networks involving TFs and miR-126, we conducted a bioinformatic analysis using the STRING database [46]. We found that EBF1, ETS2, KLF2, and EGFL7 were highly interconnected, forming complex interaction networks with other genes that jointly contribute to shared biological processes (Figure 7a). The constructed network indicated that most of the identified protein–protein interactions were between DNA-binding proteins and TFs. Apart from *ZNF35*, all genes converged to the three first-layer Gene Ontology (GO) categories “cell differentiation” (GO:0030154), “positive regulation of gene expression” (GO:0010628), and “negative regulation of gene expression” (GO:0010629) (Figure 7b).

The same three GO annotations were also associated to 117 experimentally verified miR-126 target genes [47,48,49]: we indeed identified three main clusters of biological functions directly descending from the GO terms “cell differentiation”, “positive regulation of gene expression”, and “negative regulation of gene expression” (Figure 7c). This observation suggests a functional axis involving EBF1, ETS2, KLF2, and miR-126 in regulating essential biological processes particularly relevant to BC.

To gain an unbiased global perspective, we then performed Differential Expression Gene (DEG) analysis on the 117 miR-126 target genes using matched-paired BC samples from the TCGA-BRCA dataset. We identified 97 genes with significant differential expression, including 51 upregulated and 46 downregulated genes. The top 30 upregulated and downregulated genes are shown in the Volcano plot (Supplementary Figure S5a) and as a heatmap (Supplementary Figure S5b).

**Figure 8:**
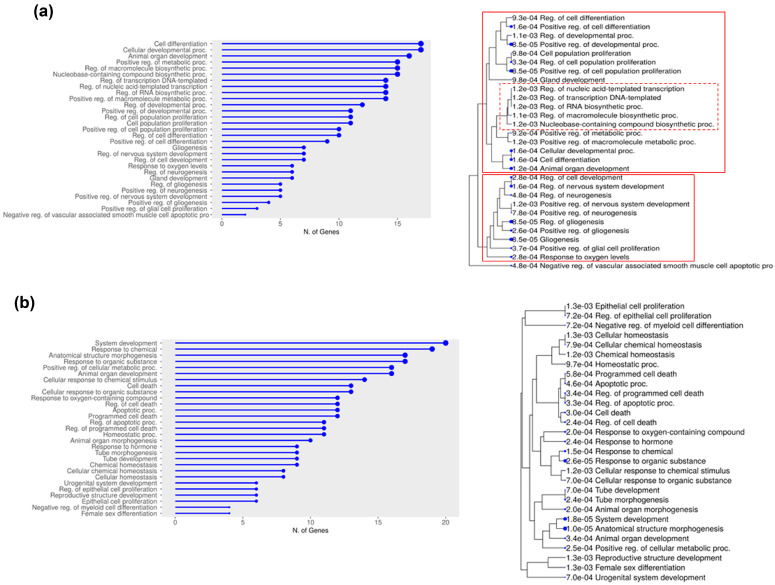

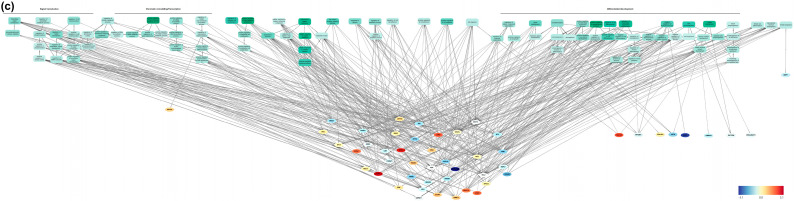
Gene Ontology (GO) analysis and network analysis of miR-126 target genes. GO and hierarchical clustering tree in “Biological Process (BP)” category for (**a**) upregulated and (**b**) downregulated miR-126 targets in TCGA-BRCA cohort, by ShinyGO. (**c**) Network analysis of miR-126 target genes in BP category, by GOnet (*p* value threshold < 5.56 × 10^−5^). Squares: categories. Circles: genes (colored by expression).

GO analysis of the overexpressed miR-126 targets revealed that nearly 60% clustered into two main biological processes (BPs) groups related to development (notably neurogenesis and gliogenesis) and proliferation/differentiation (Figure 8a). Within the proliferation/differentiation group, sixteen out of thirty genes (*E2F1*, *ATAD2*, *ZBTB41*, *SOX2*, *ZNF703*, *MYBL1*, *EZH2*, *VEGFA*, *AKT1*, *DVL3*, *PIK3R2*, *DNMT1*, *NOLC1*, *TFRC*, *BZW1*, *PGM3*) were involved in the regulation of transcription. GO analysis of the downregulated genes revealed functional relations, mainly in proliferation, cell death, homeostasis, differentiation, and development (Figure 8b).

We next dynamically explored interconnections among BPs and all DEG genes, recognizing three main annotations, concerning signal transduction, chromatin remodeling/transcription, and differentiation/development, which included miR-126 targets (Figure 8c). Specifically, twenty-nine genes belonged to the signal transduction cluster, twenty-five to the chromatin remodeling/transcription cluster, and thirty-two to the differentiation/development cluster, with thirteen genes (*VEGFA*, *DNMT1*, *ZFP36*, *PIK3R1*, *AKT1*, *SOX2*, *E2F1*, *EZH2*, *DVL3*, *ZNF703*, *TMEM100*, *MYC*, *KRAS*) shared among these clusters.

To understand why, despite the low levels of miR-126 observed, some miR-126 targets were significantly downregulated in BC (Supplementary Figure S5), we focused on EBF1/ETS2/KLF2-interacting TFs identified via STRING analysis (Figure 7a). Through interrogating several TF target gene databases [50,51,52,53], we discovered that *TAL1*, *ETS1*, *KLF5*, *PAX5*, *TCF12*, *LYL1*, *SOX7*, *SOX18*, *ETV3*, *KLF7*, *TCF3*, and *RUNX1* were transcriptional targets of EBF1/ETS2/KLF2 (Supplementary Table S1), and these TFs, along with EBF1, ETS2, and KLF2, directly controlled downregulated miR-126 target genes (Supplementary Table S2).

All these results pointed to the existence of a feed-forward loop (FFL), involving TFs, miR-126, and their gene targets, potentially playing a crucial role in tumor transformation, at least in BC.

## 3. Discussion

MiR-126 is frequently lost in malignancies, including endometrial [54], cervical [13], lung [28], pancreas [22], colon [55], and breast [15,56] cancers. Low miR-126 levels correlate with worse overall survival in lung cancer patients [57,58], while high levels improve overall survival in patients with digestive and respiratory tumors [59]. The positive role of miR-126 in cancer is further confirmed by the evidence that restoring of expression suppresses colorectal cancer growth [60] and enhances the radiosensitivity of lung adenocarcinoma [61]. Accordingly, we have previously demonstrated that miR-126 overexpression attenuates the aggressive phenotype of BC cells [14,18], while inhibition of its activity by anti-miR-126 oligonucleotide in healthy mammary cells leads to acquisition of tumor characteristics, as shown by colony-forming unit assay (Figure 3). Despite these data pointing to a potential oncosuppressive function of miR-126, the exact mechanisms underlying its deregulation in cancer remain unclear.

Altered miRNA expression in human cancers can result from multiple mechanisms, such as epigenetic alterations, defects in miRNA processing and degradation, and altered activity of specific TFs [62,63,64,65,66]. Previous studies have shown that the silencing of miR-126 expression in several tumors is due to DNA hypermethylation of the *EGFL7/miR-126* promoter regions [23,24,25,26,27,28,29,30] and histone modifications [10]. Conversely, our analysis of the epigenetic profile in BC revealed a transcriptionally active state of the *EGFL7/miR-126* gene, indicated by active histone marks (H3K4me3, H3K27ac, and H3K36m3) and DNA hypomethylation. Despite this, our BC biopsies had significantly lower pre-miR-126 levels than normal counterparts, suggesting that altered transcriptional mechanisms might be responsible for abnormal miR-126 expression.

Since the transcription of miRNAs with anti-oncogenic activity is often regulated by TFs that are themselves oncosuppressors, we focused on EBF1, ETS2, and KLF2, which are frequently deregulated in cancer [37,38,39,40,41,42], and can bind to the *EGFL7/miR-126* promoter regions. Our functional studies using RNA interference provided experimental proof of the causative relationship between these TFs and the *EGFL7/miR-126* gene, demonstrated by the drastic decrease in pre-miR-126, miR-126, and EGFL7 expression following the silencing of each TF.

Our study indicates that EBF1, ETS2, and KLF2 cooperatively act to transcribe miR-126, and their deregulation impairs miR-126 expression. Specifically, EBF1 is downregulated in BC through epigenetic silencing, as shown by high levels of H3K27me3 and DNA hypermethylation. This finding is consistent with evidence of DNA hypermethylation in primary gastric cancer [40] and cholangiocarcinoma [41]. For ETS2 and KLF2, despite promoter accessibility (as shown by H3K4me3, RNA Pol II binding, and ATAC-seq data), RNA-seq and GRO-seq profiles indicated a “poised but not transcribed” promoter state for both TFs, which may account for their downregulation in BC.

Through silencing experiments, we provided evidence that EBF1, ETS2, and KLF2 act in concert to regulate *EGFL7/miR-126* transcription. We further demonstrated that KLF2 controls ETS2 and EBF1 expression. These findings led us to hypothesize the existence of an EBF1/ETS2/KLF2/miR-126 axis. This hypothesis is supported by evidence that, despite the presence of KLF2 in some BC specimens, EGFL7/miR-126 expression was downregulated due to the loss of EBF1 and ETS2.

Deregulation of this axis may contribute to malignant transformation by profoundly affecting the expression of target genes involved in key cancer-related pathways, including growth, cellular adhesion, migration, and metastasis [16,17,18,19,67]. Key transcriptional regulators, in this context, include EZH2, DNMT1, lncRNA HOTAIR, and E2F1. Overexpression of EZH2 is related to BC progression and poor overall survival [68]. The miR-126/EZH2 axis has been described in lung adenocarcinoma, where restoring miR-126 levels inhibits EZH2, enhancing radiosensitivity, inhibiting cell migration, and promoting apoptosis [61]. Overexpression of DNMT1 is essential for silencing tumor suppressor genes via promoter hypermethylation [69]. The miR-126/DNMT1 axis is involved in azacitidine resistance in acute myeloid leukemia [70]. Upregulation of HOTAIR, promoting epithelial–mesenchymal transition, has been reported in primary BC with high metastatic potential [71]. A reciprocal negative regulation between HOTAIR and miR-126, associated with proliferation and migration, has also been found in synovial sarcoma [72]. E2F1 plays a key role in BC recurrence, proliferation, and metastasis formation [73] and is recognized as a coding driver gene in BC, responsible for epithelial–mesenchymal transition [73]. A negative correlation between miR-126 and E2F1 expression has also been found in pancreatic adenocarcinoma [74].

An in-depth bioinformatic analysis identified a novel TF-miR-126-gene FFL whose dysregulation may account for the reprogramming processes (dedifferentiation/transdifferentiation) crucial for conferring unlimited proliferation and self-renewing properties during cancer development [75]. We propose that the loss of miR-126 oncosuppressive activity in BC mainly relies on the chromatin transcriptional silencing of EBF1, ETS2, and KLF2, leading to the upregulation (through reduced miR-126 expression) and downregulation (directly and/or indirectly, by reducing the expression of other TFs) of miR-126 target genes.

This TF-miR-126-gene FFL may represent a core signal controlling the cell identity/stemness balance. Tumorigenesis is increasingly viewed as a process characterized by the acquisition of neural stemness, a state that supports cancer onset driven by ancestral gene networks promoting self-renewal and differentiation, contributing to cancer heterogeneity [76,77,78,79]. Evidence shows that cancer cells share regulatory networks with neural development. For instance, colon cancer cells express nerve cell markers and nervous system development genes [80], and cancer-related neurogenesis has been documented in human ovarian granulosa cells [81], as well as in prostate [82], bladder [83], and breast [84] cancers. The impairment of the TF-miR-126-gene FFL may cause the loss of cell identity and gain of neural features during tumorigenesis, as supported by our finding that several upregulated DEGs belong to the ontological groups “neurogenesis” (GO:0022008), “nervous system development” (GO:0007399), and “regulation of axon guidance” (GO:1902667), closely related to the formation of neurons.

In summary, we propose a paradigm shift by uncovering a TF-miR-126-gene FFL as a core barrier to the “reverse evolution” observed in cancer, where cells lose their identity and gain stemness. Our study provides insights into the molecular mechanisms underlying miR-126 deregulation in BC and offers a broader perspective on cancer development, with far-reaching implications for understanding and treating a wide range of malignancies.

## 4. Materials and Methods

### 4.1. Cell Lines

Primary human umbilical vein endothelial cells (HUVECs) (Lonza Group Ltd., Basel, Switzerland) were grown in EGM-2 Bullet kit medium (BioWhittaker, Radnor, PA, USA), as reported [85], cultured in a humidified 5% CO_2_ atmosphere at 37 °C, and used at less than four passages. Primary human mammary epithelial cells (HMECs) (Innoprot, Derio, Spain) were grown in Mammary Epithelial Cell Medium Kit (Innoprot), in a humidified 5% CO_2_ atmosphere at 37 °C, and used at less than four passages. The luminal A subtype MCF-7 cells (ATCC HTB-22) were grown in Dulbecco’s modified Eagle’s medium (DMEM), supplemented with 10% fetal bovine serum (FBS), 100 μg/mL kanamycin, 0.1 mg/mL sodium pyruvate (Biowest, Round Rock, TX, USA), and cultured in a humidified 5% CO_2_ atmosphere at 37 °C.

Cells were regularly tested for the absence of mycoplasma by using either MycoStrip™—Mycoplasma Detection Kit (InvivoGen, Toulouse, France) or LookOut^®^ Mycoplasma qPCR Detection Kit (Sigma Aldrich, Sant Luis, MO, USA).

### 4.2. Human BC Biopsy Collection

Human biopsies, including tumor and relatively healthy counterparts, were collected from six BC patients admitted at Policlinico Tor Vergata, University of Rome. No patients received any neoadjuvant (chemotherapy, hormonal, radiotherapy) therapy at the time of biopsy collection. Pathological variables, including histological tumor type, grading, stage, and immunophenotype of samples are illustrated in Table 1. This study was performed in line with the principles of the Declaration of Helsinki. Study design was carried out according to the protocol approved by the Ethical Committee of Policlinico Tor Vergata, University of Rome (reference number # 120–23). Prior to undergoing surgical procedures, all patients provided informed consent.

### 4.3. In Vitro Transient Transfections

HUVEC cells were seeded in a 6-well plate (3.5 × 10^4^ cells/well) the day before transfection. Cells were transfected with 25 nM EBF1 (Hs_EBF1#1 cod. SI04167919, Hs_EBF1#2 cod. SI04211634, Hs_EBF1#3 cod. SI04227888, Hs_EBF1#4 cod. SI04275432; Qiagen, Germantown, MD, USA), KLF2 (Hs_KLF2#4 cod. SI00463197, Hs_KLF2#5 cod. SI03162124, Hs_KLF2#6 cod. SI03246096, Hs_KLF2#7 cod. SI04275110; Qiagen), ETS2 (Hs_ETS2#2 cod. SI00074284, Hs_ETS2#3 cod. SI00074291, Hs_ETS2#6 cod. SI02662513, Hs_ETS2#7 cod. SI03097038; Qiagen), or scramble negative control (cod. 1027281; Qiagen), by using Lipofectamine RNAiMAX (Invitrogen, Heidelberg, Germany), according to manufacturer instructions. After 48 h, cells were harvested and processed for further experiments. Likewise, HMECs were seeded in a 6-well plate (1.5 × 10^5^ cells/well) the day before transfection. Cells were then transfected for 48 h with either Hs_EBF1#1 or Hs_ETS2#6 or Hs_KLF2#7 or scramble negative control and processed as reported above.

HMECs were also transfected with 25 nM of either scramble negative control (cod. AM17110, Thermo Fisher Scientific, Waltham, MA, USA) or miR-126 inhibitor (anti-miR-126, cod. AM17000, Thermo Fisher Scientific), by using Lipofectamine RNAiMAX (Invitrogen), according to manufacturer’s instructions. After 48 h, cells were harvested and processed for further experiments.

MCF-7 cells were seeded in a 6-well plate (5 × 10^5^ cells/well) the day before transfection. Cells were then transfected with 14 μg of either pCMV6-AC empty vector or with EBF1-expressing pCMV6-AC vector (Origene, Rockville, MD, USA), by using Lipofectamine 2000 (Invitrogen), according to manufacturer instructions. After 48 h, cells were harvested and processed for further experiments.

### 4.4. Quantitative Real-Time PCR

Total RNA was extracted by using the mirVANA miRNA isolation kit (Thermo Fisher Scientific), according to manufacturer instructions.

For miR-126 detection, 10 ng of total RNA were reverse transcribed with the TaqMan^®^ MicroRNA Reverse Transcription Kit (Thermo Fisher Scientific) and reverse transcription primers specific for miR-126 (Thermo Fisher Scientific). The reaction was performed on Applied Biosystems at 16 °C for 30 min, 42 °C for 30 min, 85 °C for 5 min.

For pre-miR-126, EBF1, KLF2, ETS2, and EGFL7 detection, 50 ng of total RNA were reverse transcribed with SuperScript™ IV Reverse Transcriptase (Thermo Fisher Scientific). The reaction was performed on Applied Biosystems at 65 °C for 5 min, 23 °C for 10 min, 55 °C for 10 min, 80 °C for 5 min, according to protocol supplied by the manufacturer.

For EGFL7, cDNA was amplified using SYBR Green PCR Master Mix (Bio-Rad, Hercules, CA, USA) with processing at 95 °C for 30 s, 95 °C for 15 s, 60 °C 1 min, for 40 cycles. The following primers were used:

EGFL7 forward primer: 5′-TCGTGCAGCGTGTGTACCAG-3′

EGFL7 reverse primer: 5′-GCGGTAGGCGGTCCTATAGATG-3′

All others cDNAs were amplified on 7500 Fast Real-Time PCR system (Thermo Fisher Scientific), by using TaqMan Universal PCR master mix (Thermo Fisher Scientific), with processing at 95 °C for 10 min, 95 °C for 15 s, and 60 °C for 1 min, for 40 cycles. Hsa-miR-126 (cat. n. 4427975), Hs01092694_m1 (EBF1), Hs07291763_gH (KLF2), Hs00232009_m1 (ETS2), Hs04273250_s1 (pre-miR-126) probes were produced by Thermo Fisher Scientific. The expression was calculated using the 2^−ΔΔCt^ method, after normalization with Ribosomal Protein L21 (RPL21), used as housekeeping for all, except for EGFL7, whose expression was normalized on RNU18S.

### 4.5. Colony-Forming Unit (CFU) Assay

Transiently transfected cells were trypsinized, seeded in six-well plates (1000 cells/well), and grown at 37 °C for 20 days. Afterwards, colonies were fixed and stained with 6% glutaraldehyde, 0.5% crystal violet solution, for 30 min. After washes with distilled water, the number of colonies was counted using an inverted phase contrast microscope (Zeiss, Rostock, Germany; 10× objective).

### 4.6. Datasets and Processing

ChIP-seq data, reported in Table 2, were visualized in UCSC Genome browser [86] and IGV [35]. DNA methylation cancer data were from Fortin and Hansen’s study [87] and files were converted into bigwig using Galaxy platform version 24.1.4. [88].

Transcriptomic TCGA-BRCA data, as well as pathological features of patients, were downloaded from the Data Analysis Center Firehose (http://firebrowse.org/). RNA-seq (Illumina HiSeq RNA-seq v2 Level 3 RSEM normalized expression data) of 1205 (112 normal and 1093 tumor) samples, and miRNA-seq (miRseq Preprocess Level 3 RPKM) of 1182 (104 normal and 1078 tumor) samples were used for gene expression analysis. For subsequent analyses, we identified 112 matched-paired luminal BC samples and, among them, we selected a sub-cohort of 73 BC patients (age at diagnosis: 57 ± 2 years), for whom both mRNA-seq and miRNA-seq data were available. Clinical information of 73 luminal BC patients and their tumor characteristics is provided in Table 3.

DEG analysis of 117 experimentally verified miR-126 target genes, identified by miRTargetLink 2.0 [49] and miRPathDB 2.0 [48] databases, was performed by using eVitta tool v1.3.1 [95], with a *p* value < 0.05 and Log_2_ FC threshold ≥ 0.5 or ≤−0.5.

Principal component analysis was performed by using SRplot tool [96]. GO analysis was carried out through ShinyGO v0.80 [97], Gonet v2019-07-01 [98], and miRTargetLink 2.0 [49]. TF network analysis was performed using STRING tool v12.0 [46]. Identification of experimentally verified TFs regulating miR-126 target genes was performed using hTFtarget [50], KnockTF v2.0 [51], TF2DNA [52], and TFBS [53] tools.

### 4.7. Statistical Analysis

Statistical analysis was carried out by using GraphPad Prism version 9.4.1 for Windows (GraphPad Software, San Diego, CA, USA). Mann–Whitney and Wilcoxon matched-pairs signed rank tests were used for the analysis of unpaired and paired data, respectively. The Dunnett test was used for statistical analysis of multiple comparisons. Pearson’s test was used for gene correlation analysis. Differences between geometric means were evaluated by ratio paired *t*-test. Differences were considered statistically significant at *p* < 0.05.

## Figures and Tables

**Table 1:** Clinical data of breast cancer biopsies collected at Policlinico Tor Vergata of Rome.

Case ID	Sex	Histological Subtype	Grade	UICC/AJCC Staging System ^a^	Immunophenotype
1	F	IDC	3	T4bNx	Luminal B-like
2	F	IDC	2	T1cN2a	Luminal A-like
3	F	IDC	3	T1cN1a	Luminal B-like
4	F	IDC	2	T2N0	Luminal A-like
5	F	Multifocal IDC associated with in-situ ductal carcinoma	2	T1b	Luminal A-like
6	F	IDC	2	T2N1a	Luminal A-like

^a^ UICC/AJCC 8th ed. UICC: Union for International Cancer Control. AJCC: American Joint Committee on Cancer; IDC: infiltrating ductal carcinoma.

**Table 2:** Datasets used in this paper.

Sample	Database	ID Code	Chip-Seq	Genome Annotation	File Format	Reference
Normal breast epithelium	ENCODE	ENCFF216DKX ENCFF715TWI ENCFF984VUL ENCFF529OVO	H3K4me3 H3K27me3 H3K27ac H3K36me3	19	bw	[89]
		ENCFF865DIX ENCFF490YPP ENCFF989ARX	RNA Pol II RNA-seq	38	bw	
		ENCFF570ZGR	DNAse	19	bw	
	GEO	GSM999418	DNA Methylation	19	bed	
Breast cancer (T4TD) cells	GEO	GSE107176	H3K4me3 H3K27me3 H3K27ac H3K36me3	19	bw	[90]
		GSE120162	RNA Pol II RNA-seq	38	bw	[91]
		GSE128460	GRO-seq, ATAC-seq	19	bw	[92]
KLF2	GEO	GSM3498803	Binding site	19	bedgraph	[89]
EBF1		GSM803386			bw	
ETS2		GSE127390				
HUVEC cells	GEO	GSE29611	H3K4me3 H3K4me1 H3K9ac H3K27ac H3K36me3 H3K9me3 H3K27me3 RNA Pol II	19	bw	[89]
		GSM3813846	RNA-seq	19	bw	[93,94]
		GSM2486804	GRO-seq		bedgraph	

**Table 3:** Clinical data of luminal breast cancer patients from TCGA-BRCA database.

			Amount	%
Gender		♂	1	1
		♀	72	99
AJCC stage	Grade	1	11	15
		2	38	52
		3	22	30
		4	2	3
AJCC TNM staging system	T	1	16	22
		2	43	59
		3	8	11
		4	6	8
	N	0	29	40
		1	30	41
		2	11	15
		3	2	3
		X ^a^	1	1
	M	0	68	93
		1	2	3
		X ^b^	3	4
Histological type		IDC	58	79
		ILC	5	7
		other	10	14
Immuno-phenotype		Lum A	51	70
		Lum B	22	30
Radiation therapy		Yes	28	38
		No	25	34
		NA	20	28
Neoadjuvant therapy		No	73	100
		Yes	0	0

^a^ cancer in nearby lymph nodes cannot be measured; ^b^ metastasis cannot be measured. AJCC: American Joint Committee on Cancer; BC: breast cancer; IDC: infiltrating ductal carcinoma; ILC: infiltrating lobular carcinoma; Lum: Luminal; M: presence or absence of distant metastasis; N: lymph node involvement; NA: not available; T: tumor size and extent of tumors; TCGA-BRCA: The Cancer Genome Atlas: Breast Invasive Carcinoma; TNM: tumor-node-metastasis.

## Data Availability

Data is contained within the article and Supplementary Materials.

## Supplementary Materials

The following supporting information can be downloaded at: https://www.mdpi.com/article/10.3390/ijms26010328/s1.
