# Supplementary material for: Identification of the EBF1/ETS2/KLF2-miR-126-Gene Feed-Forward Loop in Breast Carcinogenesis and Stemness
Source: Int J Mol Sci. 2025 Jan 2;26(1):328. doi: 10.3390/ijms26010328 (PMC11719960; doi:10.3390/ijms26010328)
Supplement: Supplementary file 1 [file ijms-26-00328-s001.zip › ijms-3363126-supplementary.pdf]

Supplementary information

IDENTIFICATION OF THE EBF1/ETS2/KLF2-MIR-126-GENE FEED-FORWARD LOOP IN BREAST CARCINOGENESIS AND STEMNESS

Alessandra Gambacurta[1,2], Valentina Tullio[1], Isabella Savini[1], Alessandro Mauriello[1], Maria Valeria Catani[1,#*] and Valeria Gasperi[1,#*]

[1]Department of Experimental Medicine, Tor Vergata University of Rome, Rome, Italy; gambacur@uniroma2.it; valentinatullio.nu@gmail.com; savini@uniroma2.it; alessandro.mauriello@uniroma2.it; catani@uniroma2.it; gasperi@med.uniroma2.it
[2]NAST Centre (Nanoscience & Nanotechnology & Innovative Instrumentation), Rome, Italy; gambacur@uniroma2.it
*Correspondence: gasperi@med.uniroma2.it (V.G.); catani@uniroma2.it (M.V.C.)
#Senior author

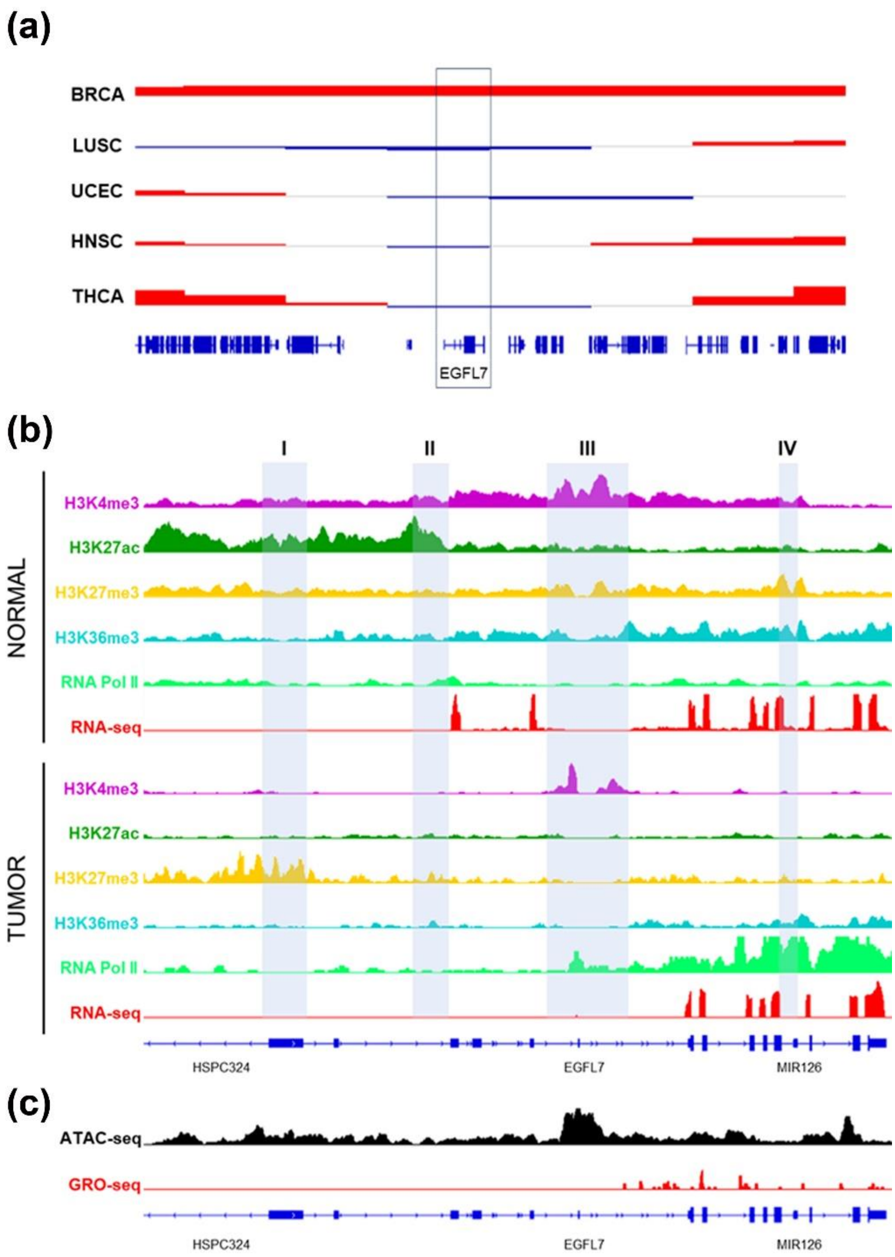

Supplementary Figure S1. Epigenetic landscape of the *EGFL7/miR-126* gene. **(a)** IGV tracks showing DNA methylation in the genome region containing the *EGFL7/miR-126* gene (square). Red: unmethylated DNA. Blue: methylated DNA. BRCA: breast cancer. LUSC: lung squamous cell carcinoma. UCEC: uterine corpus endometrial carcinoma. HNSC: head and neck squamous cell carcinoma. THCA: thyroid carcinoma. **(b)** IGV tracks showing histone modifications associated with active (H3K4me3, H3K27ac and H3K36me3) and repressed (H3K27me3) transcription, RNA Pol II occupancy and RNA-seq, in the promoter regions (I-IV) of the *EGFL7/miR-126* gene (highlighted in light blue), in normal and tumour luminal breast cells. Promoter I - chr9:139,550,026-139,555,125; promoter II - chr9:139,556,392-139,557,631; promoter III - chr9:139,559,528-139,560,398; promoter IV - chr9:139,564,447-139,565,326. **(c)** IGV tracks showing ATAC-seq and GRO-seq in tumour luminal breast cells.

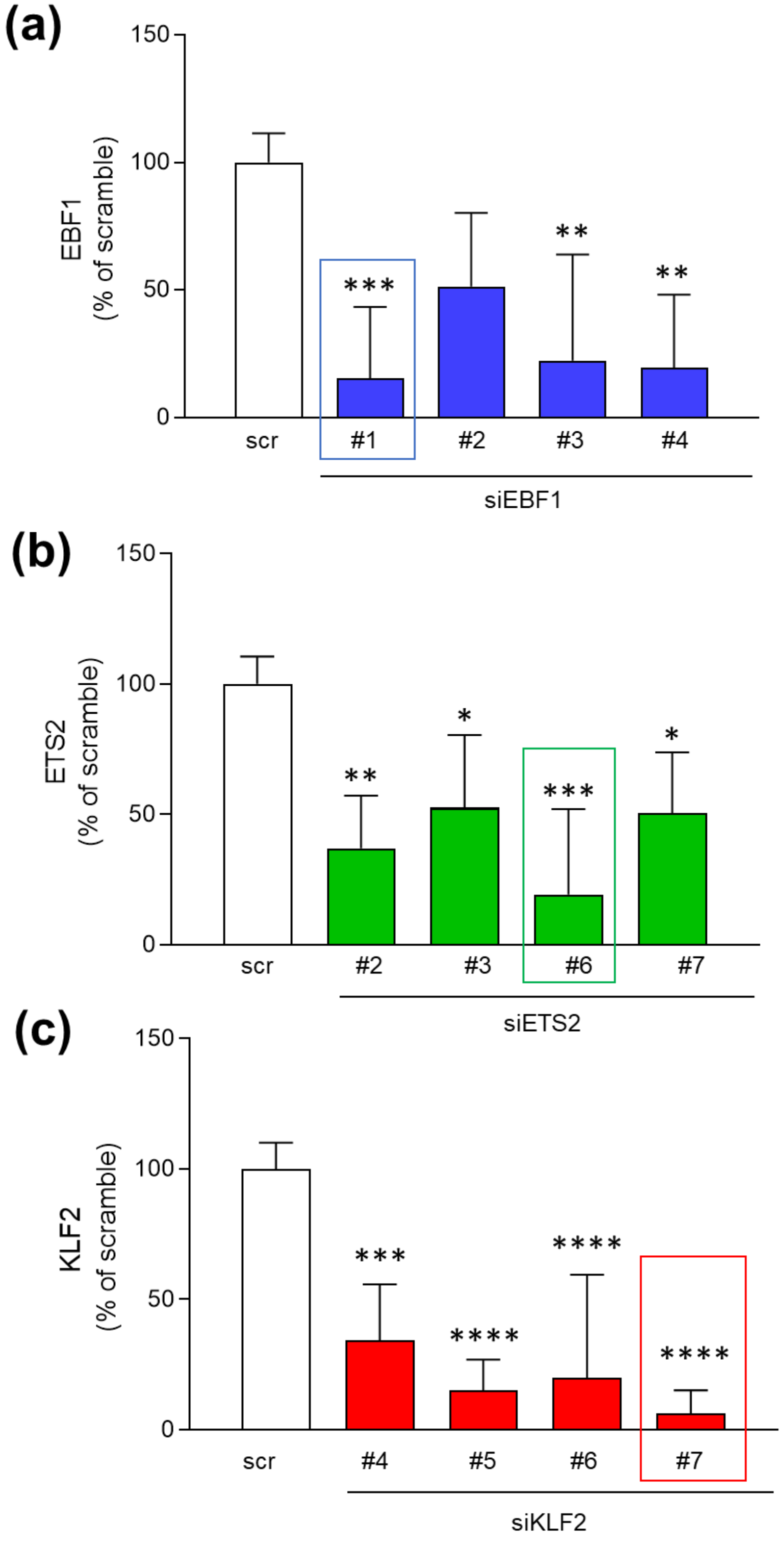

Supplementary Figure S2. Transfection efficiency of four siRNAs specific for EBF1, ETS2 and KLF2. **(a)** EBF1, **(b)** ETS2 and **(c)** KLF2 expression analysis, by qRT-PCR, after 48 h HUVEC transient transfection with four commercially available small interfering RNAs (siRNAs), specific for each transcription factor. Coloured boxes indicate the oligos chosen for subsequent experiments. Values are reported as percentage of scramble-transfected cells (scr), arbitrarily set to 100%. Data are shown as mean ± S.D. of three independent experiments, each performed in triplicate. * p<0.05, ** p<0.01, *** p<0.001 and **** p<0.0001 *versus* scramble, calculated by Dunnett's multiple comparisons test.

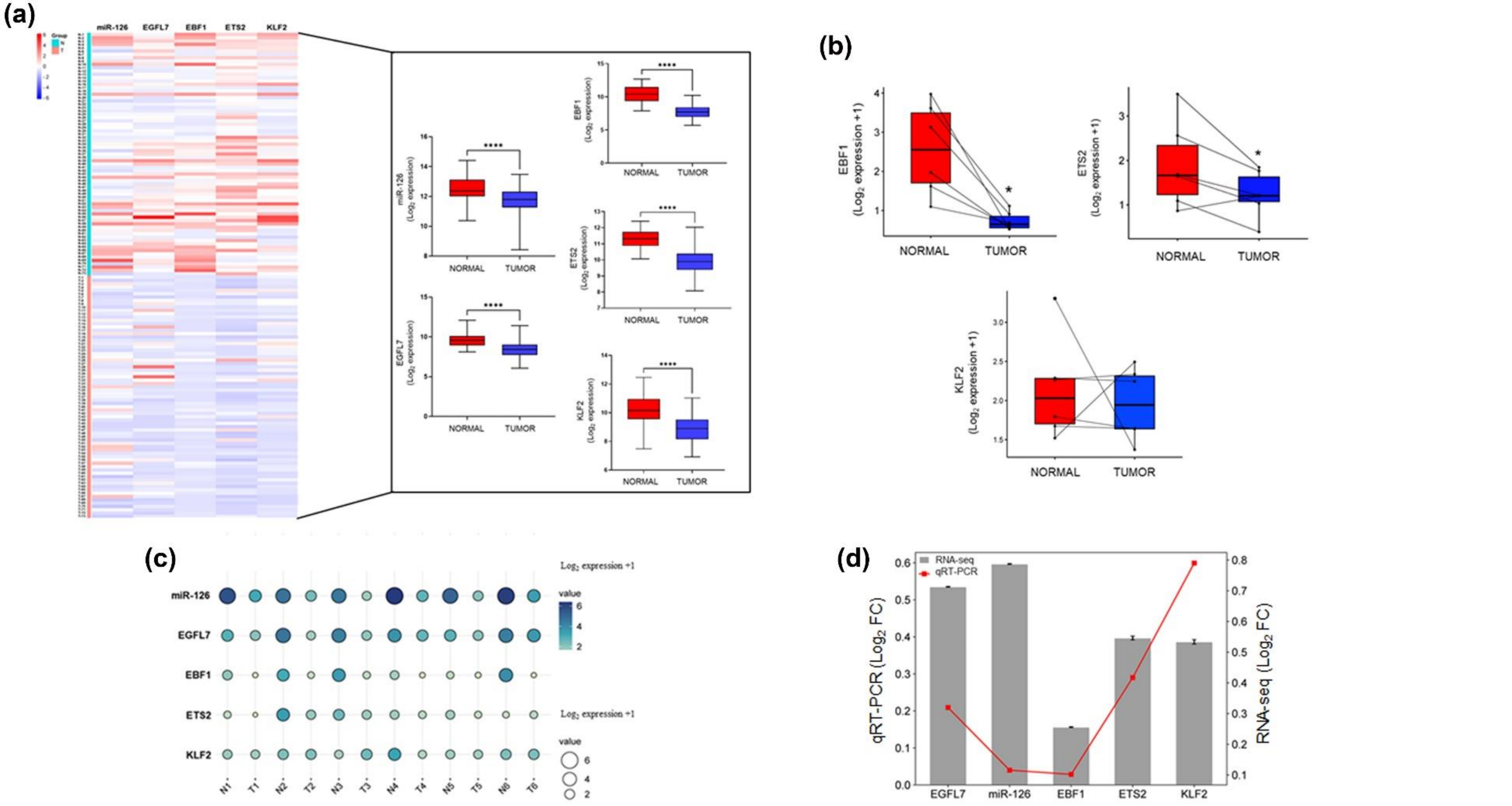

Supplementary Figure S3. EBF1, ETS2 and KLF2 downregulation in breast cancer. **(a)** Heatmap of miR-126, EGFL7, EBF1, ETS2 and KLF2 expression, in matched normal and BC samples (n=73) derived from TCGA-BRCA dataset (red for high expression value and blue for low expression value). Overall gene expression in normal and breast tumour samples is shown in the insert. Each box plot with the whiskers indicates the median, maximum and minimum expression value, and data are reported as Log_2 expression. **** $p<0.0001$ *versus* normal tissues, calculated by Wilcoxon matched-pairs signed rank test. **(b)** EBF1, ETS2 and KLF2 expression analysis, by qRT-PCR, in normal and tumour tissues derived from our cohort patients (n=6). Each box plot with the whiskers indicates the median, maximum, and minimum expression value, and data are reported as Log_2 expression+1. Solid black lines illustrate individual value expression for each patient. * $p<0.05$ *versus* matched-normal tissue, calculated by Wilcoxon matched-pairs signed rank test. **(c)** Balloon plot of gene expression levels in normal (N) and tumour (T) tissues, derived from our cohort patients (n=6). Balloon size and colon intensity refer to Log_2 expression+1. **(d)** Dual Y axis plot showing Log_2 fold change (Log_2 FC) of gene expression detected by qRT-PCR and RNA-seq. Results from RNA-seq are shown as bar graphs (mean ± S.E.M.) and the Log_2 FC values are shown on the right y axis; results from qRT-PCR are shown as red line graphs, and Log_2 FC values are shown on the left y axis.

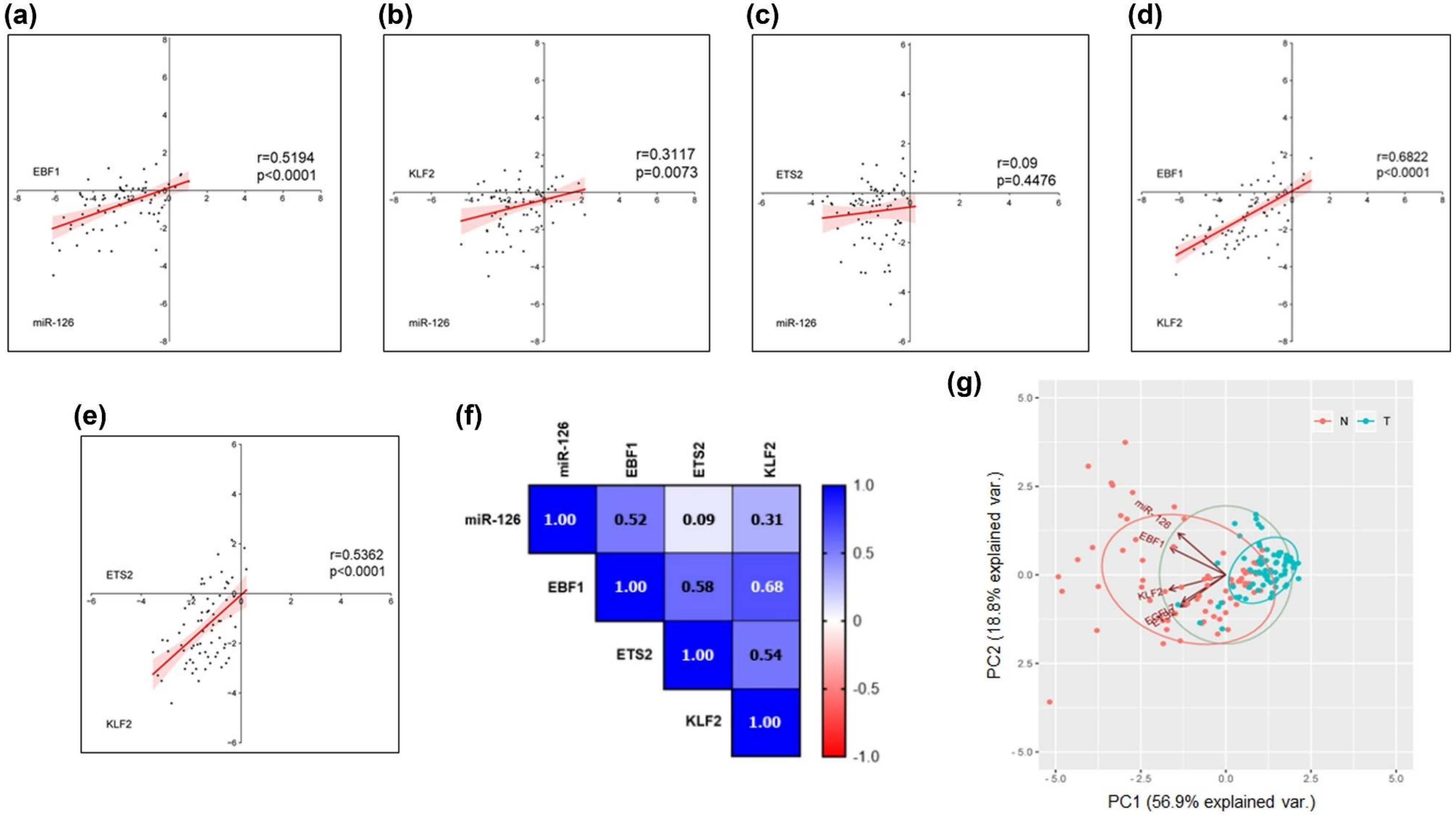

Supplementary Figure S4. Pearson correlation and Principal Component (PC) analysis. Scatter plots illustrating Pearson correlation coefficients between miR-126 and EBF1 **(a)**, KLF2 **(b)** and ETS2 **(c)**, as well as correlation coefficients between KLF2 and EBF1 **(d)** and ETS2 **(e)**. Dot plots show Log_2 fold change of gene expression (tumour *versus* matched normal samples) in TCGA-BRCA cohort (n=73). Pearson correlation coefficient (r) and *p* values are reported in each graph. **(f)** Correlation matrix plot summarizing the correlation coefficients among genes. Blue colour: positive correlation. Red colour: negative correlation. **(g)** PC analysis of TCGA-BRCA data (n=73) showing the loading of each variable (genes: brown arrows) and the scores of each patient [red points: normal (N) tissues and light blue points: tumour (T) tissues]. The length of the arrows approximates the variance of the variables, whereas the angles between them approximate their correlations.

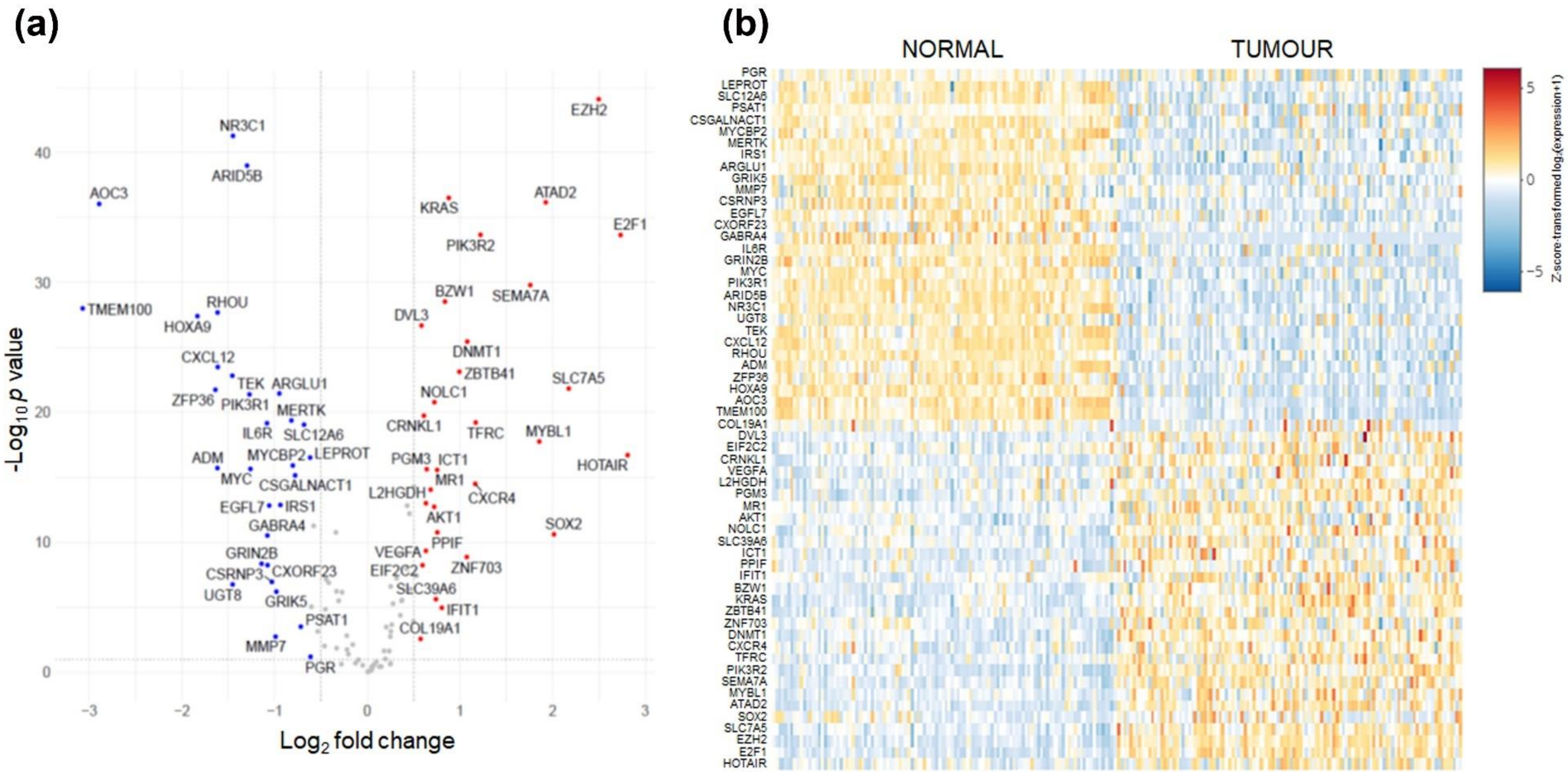

Supplementary Figure S5. Differential Expression Gene analysis of RNA-seq data in breast matched normal and cancer samples from TCGA-BRCA. **(a)** Volcano plot (fold changes versus p values) and **(b)** heatmap of the top 30 upregulated and downregulated miR-126 gene targets. Red represents upregulation and blue represents downregulation.

Supplementary Table S1. EBF1/ETS2/KLF2 control the expression of specific transcription factors.

	**EBF1**	**KLF2**	**ETS2**
EBF1		X	X
KLF2	X		
ETS2		X	
TAL1		X	X
TCF3	X	X	X
RUNX1	X		X
ETS1	X	X	X
KLF5	X	X	X
PAX5	X	X	X
TCF12	X	X	X
LYL1	X	X	X
SOX7		X	
SOX18	X	X	X
ETV3		X	
KLF7	X		

EBF1: Early B Cell Factor 1; ETS1: ETS Proto-Oncogene 1; ETS2: ETS Proto-Oncogene 2; ETV3: ETS Variant Transcription Factor 3; KLF2: Krüppel-Like Factor 2; KLF5: Krüppel-Like Factor 5; KLF7: Krüppel-Like Factor 7; LYL1: Lymphoblastic Leukemia Derived Sequence 1; PAX5: Paired Box 5; RUNX1: Runt-Related Transcription Factor 1; SOX18: SRY-Box Transcription Factor 7; SOX7: SRY-Box Transcription Factor 7; TAL1: TAL BHLH Transcription Factor 1; TCF12: Transcription Factor 12; TCF3: Transcription Factor 3.

Supplementary Table S2. Relationship between transcription factors and downregulated miR-126 target genes in TCGA-BRCA cohort.

miR-126 target gene	TF															
	EBF1[a]	KLF2[a]	ETS2[a]	TAL1[a]	TCF3[b]	RUNX1[b]	ETS1[a]	KLF5[a]	PAX5[a]	TCF12[a]	CEBPD[a]	LYL1[a]	SOX7[a]	SOX18[a]	ETV3[a]	KLF7[a]
ADM	X	X		X	X	X	X	X	X	X			X			X
AOC3		X														X
ARGLU1		X	X	X	X	X	X		X	X		X	X	X		X
ARID5B	X	X	X	X	X	X	X	X	X	X				X		X
CSGALNACT1		X		X	X	X	X		X	X	X		X			X
CSRNP3		X								X			X			X
CXCL12		X				X			X				X			X
CXorf23				X	X	X				X						
GABRA4		X											X			X
GRIK5					X	X	X	X	X	X					X	X
GRIN2B		X											X			X
HOXA9			X	X		X	X	X	X	X		X				X
IL6R	X	X		X	X	X	X	X	X	X	X		X			X
IRS1		X		X	X	X	X	X	X	X			X			X
LEPROT	X	X		X	X	X	X	X	X	X			X			X
MERTK	X	X		X	X	X	X	X	X	X			X	X		X
MMP7	X	X			X					X						X
MYC		X	X	X	X	X	X	X	X	X		X	X	X		X
MYCBP2	X	X		X	X	X	X		X	X						X
NR3C1	X	X		X	X	X	X	X	X	X	X					X
PGR		X		X		X	X						X			X
PIK3R1	X			X	X	X	X	X	X	X	X					X
PSAT1	X	X		X	X	X	X		X	X	X		X			X
RHOU		X	X	X	X	X	X	X		X			X			X

SLC12A6		X	X	X	X	X	X	X	X	X	X		X			X
TEK		X														X
TMEM100	X	X		X		X	X		X	X			X	X	X	X
UGT8		X	X	X	X	X		X	X	X			X			X
ZFP36	X	X	X	X	X	X	X	X	X	X	X					X

[a] downregulated in TCGA-BRCA cohort; [b] upregulated in TCGA-BRCA cohort.

ADM: Adrenomedullin; AOC3: Amine Oxidase Copper Containing 3; ARGLU1: Arginine and Glutamate Rich Protein 1; ARID5B: AT-Rich Interaction Domain 5B; CEBPD: CCAAT Enhancer Binding Protein Delta; CSGALNACT1: Chondroitin Sulfate *N*-Acetylgalactosaminyltransferase 1; CSRNP3: Cysteine and Serine Rich Nuclear Protein 3; CXCL12: C-X-C Motif Chemokine Ligand 12; CXorf23: Chromosome X Open Reading Frame 23; EBF1: Early B Cell Factor 1; ETS1: ETS Proto-Oncogene 1; ETS2: ETS Proto-Oncogene 2 ; ETV3: ETS Variant Transcription Factor 3; GABRA4: Gamma-Aminobutyric Acid Type A Receptor Alpha4 Subunit; GRIK5: Glutamate Ionotropic Receptor Kainate Type Subunit 5; GRIN2B: Glutamate Ionotropic Receptor NMDA Type Subunit 2B; HOXA9: Homeobox A9; IL6R: Interleukin 6 Receptor; IRS1: Insulin Receptor Substrate 1; KLF2: Krüppel-Like Factor 2; KLF5: Krüppel-Like Factor 5; KLF7: Krüppel-Like Factor 7; LEPROT: Leptin Receptor Overlapping Transcript Protein; LYL1: Lymphoblastic Leukemia Derived Sequence 1; MERTK: MER Proto-Oncogene; MMP7: Matrix Metallopeptidase 7; MYC: MYC Proto-Oncogene, BHLH Transcription Factor; MYCBP2: MYC Binding Protein 2; NR3C1: Nuclear Receptor Subfamily 3 Group C Member 1; PAX5: Paired Box 5; PGR: Progesterone Receptor; PIK3R1: phosphoinositide-3-Kinase Regulatory Subunit 1; PSAT1: Phosphoserine Aminotransferase 1; RHOU: Ras Homolog Family Member U; RUNX1: Runt-Related Transcription Factor 1; SLC12A6: Solute Carrier Family 12 Member 6 ; SOX18: SRY-Box Transcription Factor 18; SOX7: SRY-Box Transcription Factor 7; TAL1: TAL BHLH Transcription Factor 1; TCF12: Transcription Factor 12; TCF3: Transcription Factor 3; TEK: TEK Receptor Tyrosine Kinase; TF: Transcription Factor; TMEM100: Transmembrane Protein 100; UGT8: UDP-Glucuronosyltransferase 8; ZFP36: Zinc Finger Protein 36.
